# European springtime temperature synchronises ibex horn growth across the eastern Swiss Alps

**DOI:** 10.1111/ele.12231

**Published:** 2013-12-16

**Authors:** Ulf Büntgen, Andrew Liebhold, Hannes Jenny, Atle Mysterud, Simon Egli, Daniel Nievergelt, Nils C Stenseth, Kurt Bollmann

**Affiliations:** 1 Swiss Federal Institute for Forest Snow and Landscape Research (WSL)Birmensdorf, CH-8903, Switzerland; 2 Oeschger Centre for Climate Change Research (OCCR), University of BernBern, CH-3012, Switzerland; 3 Global Change Research Centre AS CR, v.v.i.Bělidla 986/4a, Brno, CZ-60300, Czech Republic; 4 Northern Research Station, USDA Forest ServiceMorgantown, WV, 26505, USA; 5 Department of Wildlife and Fishery Service GrisonCH-7001, Chur, Switzerland; 6 Centre for Ecological and Evolutionary Synthesis (CEES), Department of Biosciences, University of OsloPO Box NO-1066, Blindern, 0316, Oslo, Norway

**Keywords:** Alpine ungulates, body size, climate change, ecological response, European Alps, horn growth, phenotypic plasticity, plant phenology, spatial synchrony, trophic interaction

## Abstract

Direct effects of climate change on animal physiology, and indirect impacts from disruption of seasonal synchrony and breakdown of trophic interactions are particularly severe in Arctic and Alpine ecosystems. Unravelling biotic from abiotic drivers, however, remains challenging because high-resolution animal population data are often limited in space and time. Here, we show that variation in annual horn growth (an indirect proxy for individual performance) of 8043 male Alpine ibex (*Capra ibex*) over the past four decades is well synchronised among eight disjunct colonies in the eastern Swiss Alps. Elevated March to May temperatures, causing premature melting of Alpine snowcover, earlier plant phenology and subsequent improvement of ibex food resources, fuelled annual horn growth. These results reveal dependency of local trophic interactions on large-scale climate dynamics, and provide evidence that declining herbivore performance is not a universal response to global warming even for high-altitude populations that are also harvested.

## Introduction

Increasing temperatures and shifts in precipitation regimes, along with complex ecological responses have been extensively reported at various spatiotemporal scales throughout the world. However, such environmental changes are particularly severe for arctic and alpine ecosystems (Post *et al.* 2009; Pauli *et al.* 2012), where modification in climatological means and extremes may be strong enough to disrupt trophic interactions and the seasonal synchronisation of interdependent ecological processes (Ims *et al.* 2008; Post *et al.* 2008; Hansen *et al.* 2013).

In addition to shifts in population distribution, phenology, behaviour and even extinction of many species (Parmesan & Yohe 2003; Thomas *et al.* 2004; Fordham *et al.* 2013), impacts on body size and physiology associated with changing temperatures have been observed in several organisms (Ozgul *et al.* 2010; Gardner *et al.* 2011; Tafani *et al.* 2013). Unravelling biotic from abiotic drivers of such changes is, however, fraught with difficulties because the spatial and temporal extents of animal population data are generally limited (see references herein). Shrinking body size, as a direct response to environmental conditions, can influence thermal biology, as well as energy and homeostatic balance, both of which potentially influence a species' resilience to warmer temperatures (Both *et al.* 2006). Phenotypic plasticity and evolutionary adaptability play a key role in determining whether a species can tolerate the speed and magnitude of ongoing environmental changes (Hoffmann & Sgrò 2011), especially for taxa that live near their physiological limits and are unable to disperse to more suitable climates, or are adapted to small ecological niches that are spatially constrained within specific elevational limits.

Strong selection for secondary sexual characteristics (Monteith *et al.* 2013), such as horns and antlers, exists in many species and production of such structures is dependent on the availability of the same resources, which are allocated to other traits that directly impact fitness (Toïgo *et al.* 2007). Consequently, the size of horns and antlers is closely related to an animal's overall performance (Robinson *et al.* 2006; Bergeron *et al.* 2010), and likely also appears sensitive to direct and indirect external forcing factors, including food availability and climate variability (Festa-Bianchet *et al.* 2004), as well as the genetic architecture (i.e. the number of genes and the magnitude of their effects) of trait variation (Johnston *et al.* 2013).

Horn growth in male bovines, long known to have arisen via sexual selection, thus represents a unique biological indicator of past environmental conditions (von Hardenberg *et al.* 2007; Bergeron *et al.* 2008). In fact, accurate measurements of yearly horn increments that are possible for many species provide a rich environmental archive (Giacometti *et al.* 2002), somewhat comparable to the yearly resolved information on temperature and precipitation that is often preserved in extra-tropical tree rings (Büntgen *et al.* 2011). Particularly wide annual increments with distinct boundaries are characteristic features of the large horns produced by male Alpine ibex (*Capra ibex*) (see Figs2). These growth increments, which can accumulate in horns > 1 meter in length, integrate effects of chronological age, climate-induced food quality and quantity, intraspecific competition, maternal feeding and genotypic constitution, for instance (Giacometti *et al.* 2002; Barbosa *et al.* 2010).

**Figure 1:**
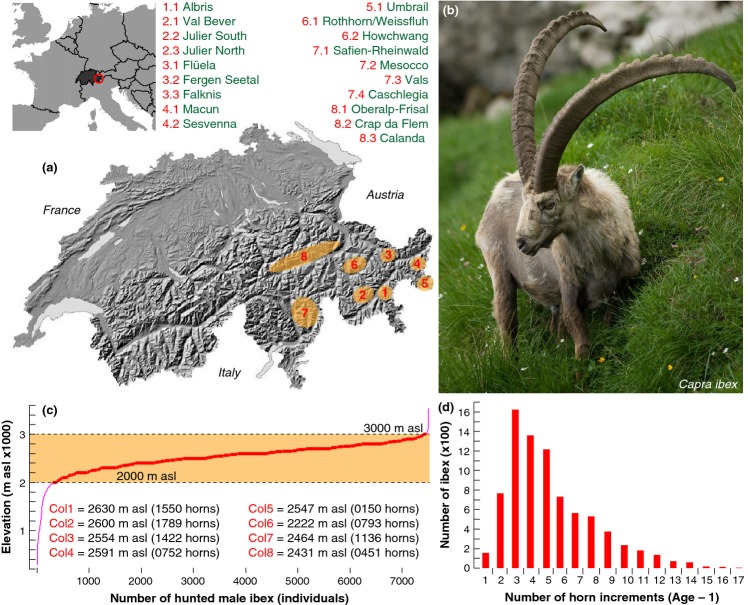
Description of the ibex data set. (a) Location of the eight main ibex colonies in the eastern Swiss Alps. (b) An old male ibex in its typical Alpine environment with distinct annual horn growth increments (Photo: J. Senn; WSL). (c) Cumulative elevational distribution of ibex hunting locations (Figure S2) including the mean hunting elevation per colony that ranges from 2222 m asl in colony 6 to 2630 m asl in colony 1 (number in parenthesis refers to the number of hunted animals per colony). (d) Distribution of 42 239 annual horn increments from 8043 individual ibex indicates that the majority of animals have 3–5 growth rings, and thus were 4–6 years old when hunted.

**Figure 2:**
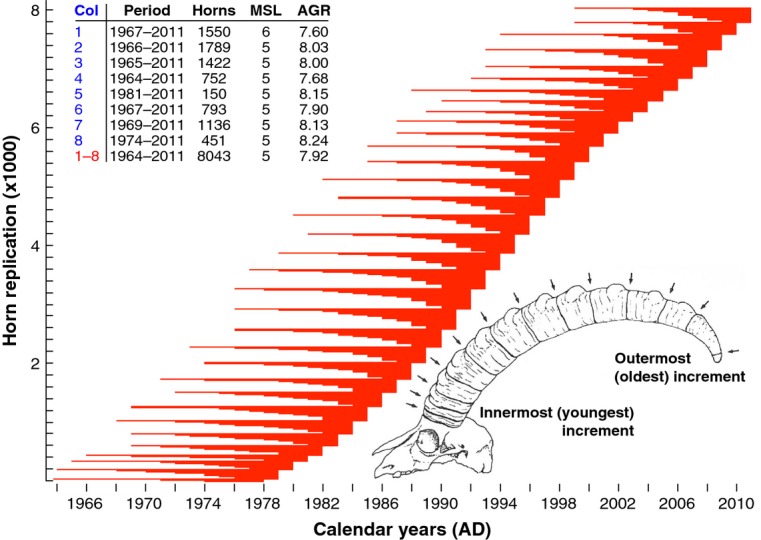
Horn series replication. Distribution of 8043 horn measurement series with their individual start and end dates ranging from 1964 to 2011. The data set comprises 42 239 well-defined annual growth increments, and the inset table provides characteristics for data from each colony: Mean segment length (MSL) refers to the mean number of annual growth increments per horn, whereas average growth rate (AGR) indicates the average increment width per colony. The sketch describes a horn structure with 12 discrete increments (kindly obtained from R. Zingg). The outermost (innermost) segment represents the oldest (youngest) increment, i.e. refers to the year of birth (hunting).

Here, we investigate how variation in European climate influences annual horn growth rates of male Alpine ibex at eight disjunct colonies in the eastern Swiss Alps. Dendroclimatological time-series analyses are, for the first time, applied to horn length data of this charismatic species, along with information on the precise location, body weight and hind-foot length recorded for each sampled specimen, as well as to meteorological and plant phenological observations. We further use this unique interdisciplinary approach to assess the dependency of local trophic interactions on large-scale climate dynamics, and to detect warming-induced phenotypic plasticity.

## Material and Methods

### Horn growth measurements

We measured a total of 42 239 annual horn growth increments from 8043 male Alpine ibex sampled at eight geographically distinct colonies in the eastern Swiss Alps (Fig. 1a,b). All animals were shot by locally registered hunters under the direction of a government game warden, as part of a unique and long-standing environmental protection system in the Canton Grison (www.jagd-fischerei.gr.ch). The vast majority of all animals were shot between 2000 and 3000 m asl (Fig. 1c), with the mean hunting elevation per colony ranging from 2222 to 2630 m asl, and little changes over time. Average hunting elevation before and after 1995 was 2500 and 2533 m asl respectively. The number of animals sampled per colony varied considerably from 150 to 1789 (Fig. 1c). For each animal, the total horn length and the length of each annual increment were precisely measured at the outer lateral side of the longer horn following a standardised protocol (Ratti 1981, 1994; Buchli & Abderhalden 1998). The first annual increment (Figs1b, 2), i.e. the oldest and outermost horn segment was excluded from each series to avoid biases arising from abrasion and possible maternal effects (Giacometti *et al.* 2002). Most ibex had 3–5 growth increments per horn and thus were 4–6 years old when hunted (Fig. 1d); more than ten segments were only found for a few specimens. Along with individual horn growth, hunting location, animal age, body weight and hind-foot length were recorded for each male ibex (see supporting online information).

### Horn growth chronologies

The individual start and end dates of the 8043 series of horn measurements that comprise 42 239 well-defined growth increments range from AD 1964–2011 (Fig. 2). This extremely uniform temporal sample distribution, along with the annual resolution and large sample size makes these data suitable for the application of time-series analyses commonly used in tree-ring research (i.e. dendroclimatology and dendroecology).

As a first step, horn growth data were compiled for each population in the TUCSON-system, a standard structure for organising tree-ring data and subsequently processing them via the ARSTAN-software (http://www.ldeo.columbia.edu/tree-ring-laboratory). Additionally, we separated all horn data into 3911 short (young) and 4132 long (old) series to assess possible age-related differences in their growth behaviour. The number of annual horn increments in the short (long) data set ranged from 1 to 4 (5–17) between 1975 and 2011 (1964 and 2011), with a mean growth rate of 8.5 (7.4) cm year^−1^. Data were also divided into groups of higher and lower elevations (</>2550 m asl) to explore potential effects of elevation on horn growth. The resulting low-elevation chronology comprised 2530 horn series from colonies 5–8, had a mean growth rate of 8.1 cm year^−1^, and continuously covered the 1967–2001 period. The high-elevation chronology, containing 5513 horn series between 1964 and 2001, was better replicated and had a slightly lower mean growth rate of 7.6 cm year^−1^.

Horn growth series from each colony were then age-aligned to assess the mean non-climatic, bio-physiological growth trend that is characteristic for each of the eight populations (see also supporting information). A similar trial was performed for the different age and elevational subsets. The resulting growth functions (Figure S1), the so-called Regional Curves (RCs), were then used to remove age trends from the raw horn measurements via application of the regional curve standardisation method (RCS; see references in the supporting online information). This age-related, composite detrending technique is commonly used for (tree) growth-climate response analyses, and thus describes an essential component of (tree ring-based) climate reconstructions (Büntgen *et al.* 2011). In fact, RCS allows putative high- to low-frequency information to be preserved, and is accomplished in three steps: (1) All measurement series are aligned by their first year of growth. (2) The mean of all age-aligned series, the RC is smoothed using a cubic spline of 10% the series length (supporting information). (3) Deviations of the individual measurements from this smoothed RC, referenced by calendar dates, are expressed as ratios between the original and fitted values. The detrended and calendar dated individual horn growth values were averaged using the bi-weight robust mean, the variance in these chronologies was additionally stabilised, and bootstrapping was ultimately performed to define confidence intervals (see also references in the supporting online information). In addition to application at the population, elevation and age-class level, the RCS detrending method was also employed for the entire data set pooled across all 8043 individual horn measurement series.

### Horn growth responses

The resulting grand average horn growth chronology was correlated against a variety of environmental factors, whereas the eight colony-specific chronologies were utilised to measure the degree of spatial synchrony in annual horn growth, which was quantified by the nonparametric spatial covariance function (supporting information). In this method, synchrony is characterised by a correlogram consisting of smoothed cross-correlation between time and series relative to the distance separating the series.

Records of different climatological indices as well as plant phenology were available at monthly resolution and coincide with the 1982–2011 period during which ibex populations in the eastern Swiss Alps remained fairly stable (Giacometti *et al.* 2002). Monthly (February–June) and seasonal (between February and June) maximum temperature means (1950–2012) were drawn from a larger scale (25–75° N and 40° W–75° E), high-resolution gridded (0.25° regular latitude – longitude grid cells) data set, herein averaged over the 8–8.5° E and 46–46.5° N study region. Correlation maps were calculated via the KNMI Climate Explorer (http://climexp.knmi.nl/) to assess the spatial significance of temperature associations with horn growth. Monthly indices of the North Atlantic Oscillation (NAO), herein expressed as the pressure difference between Gibraltar and Iceland (Jones *et al.* 1997) were paired with ibex growth series.

Total and new snowfall measurements came from the Arosa station, which is situated in between the eight ibex colonies at 1840 m asl in Grison. A total of 39 daily plant phenological observations were made between mid-April (DOY; day of year 104) and mid-July (DOY 193) at five high-elevation (> 1500 m asl) stations (Davos, Zuoz, Lenzerheide, St. Moritz, Pontresina) within the study region. These observations successively span the 1970–2012 period. Comparison was also made with a newly developed tree-ring width chronology from 42 pine (*Pinus cembra*) trees. All trees were sampled within the high-elevation treeline ecotone of the Upper Engadine close to St. Moritz (*c*. 46°27′40” N, *c*. 9°46′47” E and *c*. 2100 m asl). This record covers the 1724–2011 period at a replication of > 10 series and was, comparable to the ibex horn growth measurements, processed by means of the RCS method.

### Mixed-effect models

To ensure that density dependence did not interfere with the statistical characterisation of climate effects, a linear mixed-effect model was additionally performed; annual horn segments were analysed at the individual level with ID as a random factor over the period 1976–2011 (see Tables S1–S2 for details). This period was selected because population size estimates (systematic animal counts following a standardised procedure that is unique to the Canton Grison) were available via the Department of Wildlife and Fishery Service Grison (www.jagd-fischerei.gr.ch). Given that ibex abundance is highly stationary (Giacometti *et al.* 2002; Biebach & Keller 2012) (Figure S2), though estimates may contain some degree of uncertainty (Largo *et al.* 2008), we utilise population size as a surrogate for population density (Sæther *et al.* 2007).

## Results

### Horn growth synchrony

**Figure 3:**
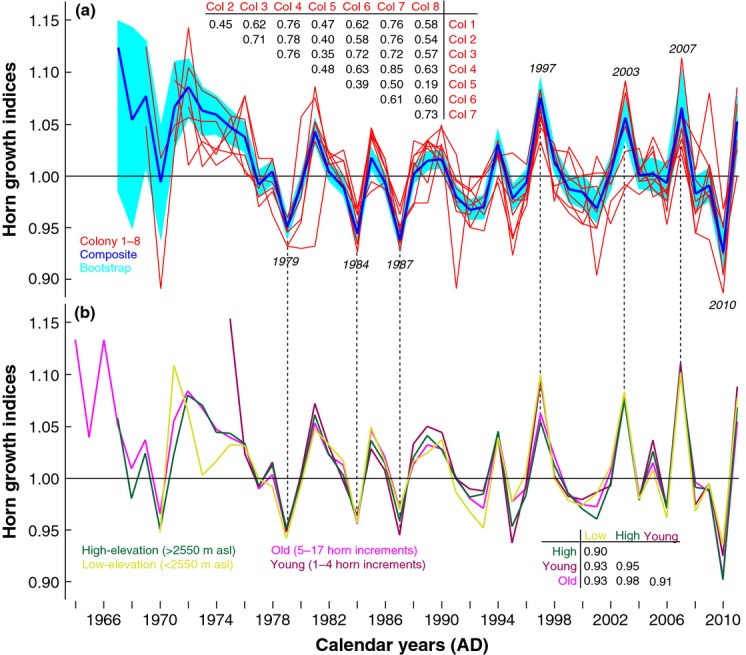
Horn growth synchrony. (a) Annual horn growth variability of the eight colonies (red) and their composite (blue) and the 95% bootstrap confidence interval (light blue). The RCS detrended chronologies are truncated at a minimum replication of ten horns. Corresponding correlation coefficients between the eight individual horn chronologies were computed over their common period. (b) Comparison between four independent horn growth chronologies using either young (3911 horns) and old (4132 horns) individuals, or low (2530 horns) and high (5513 horns) individuals. Corresponding correlation coefficients between the subset chronologies were computed over the 1982–2011 period.

A surprisingly high level of interannual horn growth variability is spatially synchronised among all eight ibex colonies within the area of 2123 km^2^ in the eastern Swiss Alps, Canton Grison (Fig. 3a). All individual horn chronologies show distinct positive growth anomalies in 1997, 2003 and 2007, whereas negative departures are most pronounced in 1979, 1984, 1987 and 2010. The common growth coherency is reflected in a highly statistically significant grand average correlation (*r = *0.77; *P < *0.001) among all colony-specific chronologies. This exceptional level of year-to-year growth synchrony is further highlighted by the remarkable agreement of two age-class chronologies solely based on either young (1–5 years) or old (6–17 years) animals (Fig. 3), as well as two elevation-class chronologies that exclusively contain data from either lower or higher elevation populations (</>2550 m asl).

The independent young and old (low and high) chronologies correlate at *r = *0.92 (0.90) over the 1982–2011 period (Fig. 3). This finding, contrasts with the overall lowest cross-chronology correlation obtained from colony # 5, which consists of only 150 horn series (Fig. 3), and emphasises the importance of sample size to the detection of synchrony, a factor that is similarly important in dendrochronology. Moreover, there also is evidence of declining horn growth synchrony with increasing distance between populations (Figure S3). Less cross-colony synchrony and increasing chronology uncertainty together with generally higher growth variability prior to *c*. 1978 coincide with an uneven distribution of series start and end dates (Fig. 2), which can be related to a typical reduction in sample size and decreasing (increasing) mean horn length (age) back in time (Figure S4).

In addition to the high degree of year-to-year growth synchrony among the eight geographically disjunct colonies as well as synchrony among the elevational subsets, absolute growth levels are nearly identical among the eight colonies and among elevations (Figure S2). This information is best reflected by the Regional Curves (RCs) that were initially employed to eliminate age trends from the raw horn measurements by means of the regional curve standardisation method (RCS). Their shapes, independent of the data subset used, almost perfectly resemble linear functions (Figure S1), whereas age trends found in tree rings usually describe negative exponential functions (see also Figure S8). It should be further noted that interannual fluctuations in colony-specific ibex horn growth and its spatial agreement are largely unaffected by differences in population size of the eight settings (Figurs S5, S6).

### Horn growth drivers

**Figure 4:**
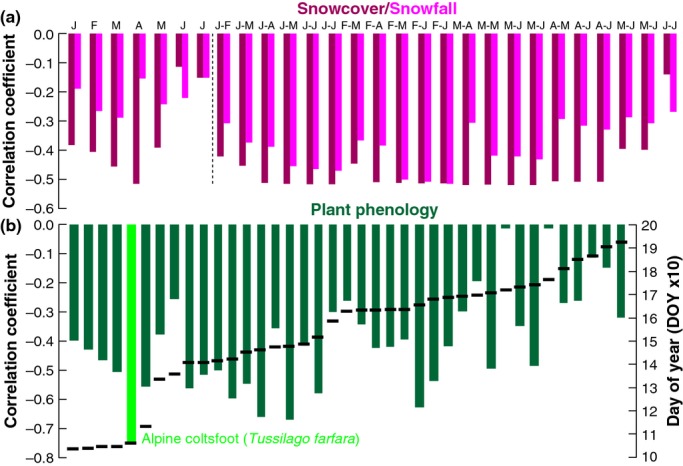
Correlations of horn growth with environmental factors. (a) Correlation coefficients (1982–2011) between the composite residual RCS horn chronology and monthly and seasonal snowcover and snowfall data between January and July, and (b) 39 different phenological records sorted by their average annual date of appearance from left to right (DOY; black dashes).

Average monthly accumulated snowcover from January to May is negatively correlated with horn growth in the same year (*P < *0.001) (Fig. 4a). The month with the strongest association is April (*r *=* *−0.52). Evaluation of actual snowfall level reveals a lower correlation but similar intraannual trends. Early season plant phenology recorded at higher elevation sites and before June (DOY < 150), which is affected by the amount and duration of snowcover, correlates negatively (*P < *0.001) with horn growth rate (Fig. 4b), whereas phenological measurements from June onwards (DOY > 150) appear to be less important. The inverse relationship between the onset of high-elevation plant growth and horn increment is best expressed by the blossoming of Alpine coltsfoot (*Tussilago farfara*), a typical perennial herbaceous in high-elevation meadows (Figure S7). The correlation between annual horn growth and coltsfoot plants from 1982 to present is 0.76 (*P < *0.001).

The assessment of 42 high-elevation pine trees reveals an age trend that resembles a negative exponential curve (Figure S8a), which is somewhat different from the age-dependent linear decline in annual horn growth increments (Figure S1). However, both biological systems are in a similar way characterised by faster juvenile and slower mature growth, a pattern that is further expressed by the relationship between mean segment length (MSL; number of rings per series) and average growth rate (AGR; mean ring width per series) – series with fewer rings exhibit generally higher growth levels (Figure S8b). Interannual to multi-decadal variability in pine growth is characterised by increasing values over most of the 20th century (Figure S9a). This finding is in good agreement with the long-term rise of Central European summer temperatures (see discussion below). It should be noted that the tree-ring chronology contains 42 series (Figure S9b), while the ibex record consists of 8043 series. Non-significant correlation (*r *=* *0.09) between the pine and ibex time-series confirms the different seasonal sensitivity of these two parameters (Figure S9c): Ibex horn growth depends on spring temperatures from March–May but pine growth reflects a clear summer temperature signal.

**Figure 5:**
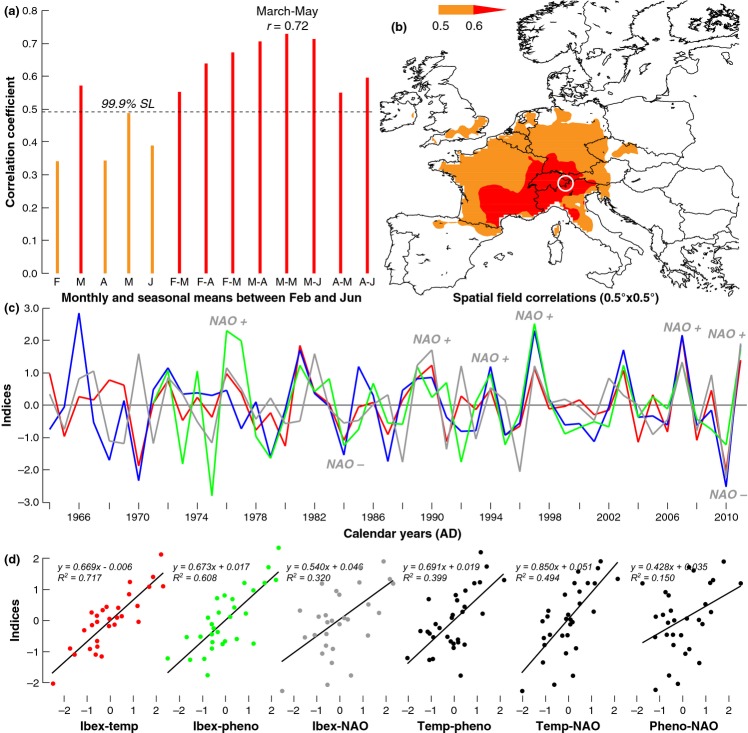
Relationships between horn growth and temperature. (a) Correlation coefficients between the composite residual RCS horn chronology and monthly and seasonal maximum Alpine temperature means (1982–2011). (b) European-wide spatial correlation field between the composite residual RCS horn chronology and March–May maximum temperatures. (c) Comparison between the composite residual RCS horn chronology (blue), March–May maximum Alpine temperatures (red), inverse springtime phenology of Alpine coltsfoot (green), and February–May NAO indices (grey). All time-series were first scaled to the corresponding March–May (MAM) maximum temperature values (anomalies in °C) back to 1982 and then 10-year high-pass filtered, with (d) showing the corresponding linear regression models of all pairings calculated over the 1982–2011 period.

The statistically significant positive correlation (*P < *0.001) between maximum spring temperature (March–May average) and ibex horn growth of the same year is indicative of the linkages between early springtime warming, productivity of water-saturated high-elevation ecosystems, plant phenology and ibex performance (Fig. 5a). The geographical extent of the observed growth-climate response (i.e. the area over which correlations are > 0.6) extends roughly over the entire Alpine arc from southern France to western Austria (Fig. 5b). An area of slightly lower correlation (0.5–0.6) covers most of Central Europe and to the northwest extending almost to the British Isles. This spatial signature suggests the importance of a dominant climate regime at the transition between winter and springtime that is mainly responsible for the transportation of warm Atlantic air masses across the continent, the NAO.

Figure 5c illustrates the congruence among the trophic interactions that are likely responsible for the distinct interannual variability and strong spatial synchrony in Alpine ibex horn growth. Synoptic-scale pressure anomalies of the NAO averaged over February to May drive springtime climate in the European Alps. The best linear mixed-effect model (random term for individual) of horn increment includes age (up to 3rd order), colony, maximum temperature (March–May), snowcover (March–May), population size and year (as trend) as main effects, and the interactions between colony and age (up to 2nd order) and colony and population size. There is a positive effect of maximum temperature and a negative effect of snowcover. As expected, population size negatively affects growth in four colonies, while estimated effects are close to zero in the three other colonies, as well as weakly, but statistically positive in the remaining colony (the baseline in Table S1). The residual effect of year (as trend) after entering other environmental variables is weakly negative, which might reflect some degree of hunting selection, but could also result from other factors (Table S2). However, note that the main effect of year (as trend) before entering environmental variables is positive [estimate = 0.00033 (0.00000, 0.000656)]. The effects of temperature and snowcover are robust to different model specifications. Using a mixed model with both ID and colony as random terms also reveals positive effects of temperature, weakly negative effects of population size and negative effects of snowcover and year (see supporting online information). Attempts to control for density dependence in horn growth rates had no impact on the reported patterns related to climate effects (Table S3).

**Figure 6:**
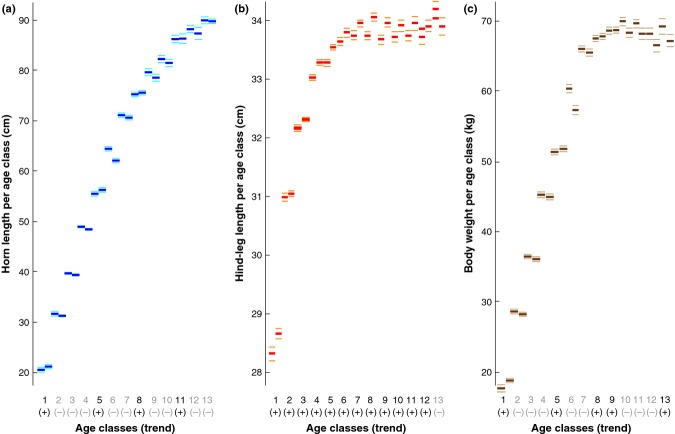
Phenotypic variation in horn length, body size and weight. (a) Mean horn length (cm), (b) mean hind-leg length (cm), and (c) mean body weight (kg) of each individual male ibex computed over two early/late split periods (1978–1994/1995–2011) and for each age classes that is replicated by > 100 horn series, i.e. age 1 to age 13. Horizontal bars refer to mean values associated with ± 1 standard error, and parentheses at the *x*-axis indicate a positive (+) or negative (−) shift over time.

Total horn length averaged over two independent early and late periods (1978–1994 and 1995–2011) shows increasing values for four age classes and decreasing values for nine age classes (Fig. 6a). Almost all changes are, however, not statistically significant and relative increment gain continues over almost the entire lifespan. Comparison of mean annual hind-foot length growth over the same split intervals and stratified by age class reveals a strong tendency for increasing body size (Fig. 6b). Positive changes in hind-foot length, which reflects body size are evident in ^12^/_13_ age classes, with statistically most significant growth increases found in seven age classes. However, no evidence was obtained for a consistent long-term alteration in body weight, a finding that is consistent among the different age classes (Fig. 6c), and in agreement with the heterogeneous patterns found for long-term changes in horn length.

## Discussion

Spatial synchrony through time is a widely observed characteristic in population traits such as animal abundance, plant seed production and tree growth (Liebhold *et al.* 2004). While in many cases, such synchrony can arise from movement of individuals, synchrony in traits such as annual horn growth and tree growth must arise from the ubiquitous presence of synchrony in weather that nearby populations are exposed to.

Anomalously high springtime temperatures promote earlier and more rapid snowmelt, which in turn accelerates and prolongs seasonal plant growth across the Alpine arc. Such early onset of vegetative growth in herbs and grasses at higher alpine elevations enhances ibex resource quality and abundance. Consequently, an upward trend in springtime temperatures should translate into increases in ibex growth. Indeed, slightly positive long-term trends are found in both meteorological statistics and in the horn growth records (Figure S9c).

The association of increased horn growth with earlier onset of vegetative growth and rising temperatures since the early-1980s (Büntgen *et al.* 2011), most likely results not only from the seasonal extension of the period of ibex resource availability, but also because earlier snowmelt and rainfall translate into increased soil moisture availability and associated increases in ibex nutritional resources. This conclusion is in agreement with evidence from other large herbivores at northern latitudes and high altitudes, where individual performance often depends directly on both springtime food availability and quality, and therefore indirectly on the prevailing climate, which affects the timing of snowcover and plant phenology (Pettorelli *et al.* 2005, 2007).

The high level of interannual horn growth variability that is spatially synchronised among all eight ibex colonies within the eastern Swiss Alps clearly exceeds the degree of coherency commonly found in Alpine tree-ring networks (Büntgen *et al.* 2008). Moreover, horn increments of similarly old male Alpine ibex from different locations and elevations exhibit comparable growth levels, and therefore contradict the putative importance of local site conditions such as geology and exposition as well as local availability of food resources (Giacometti *et al.* 2002; Barbosa *et al.* 2010). In contrast to horn growth, annual and long-term changes in population size were found to be uncorrelated among the eight colonies though population counts are subject to measurement error (Largo *et al.* 2008), which may have obscured associations.

It is also interesting to note that the decline of horn increments with age accelerates after *c*. 6–8 years, which most likely reflects the impact of sexual maturation and consequent peak reproduction that occurs around that age in this ungulate species (Willisch *et al.* 2012). The absence of a consistent age-related trend in body weight possibly reflects the overall high variability of this parameter, whereas hind-foot length is generally, but not always (Martin *et al.* 2013), considered as a more accurate measure.

Phenotypic correlates of body size (such as skeletal hind-foot length) are widely used in vertebrate studies and provide a useful indicator of eco-physiological growth conditions of immature animals (Garel *et al.* 2010), especially in large mammalian herbivores living in harsh environments with strong seasonal resource limitations. Our failure to detect a long-term trend of decreasing body size demonstrates that such a tendency is not a universal response to climate change (Gardner *et al.* 2011), even in more extreme alpine environments (Ozgul *et al.* 2010). Our results contrast with previously reported patterns of decreasing body mass in three alpine chamois (*Rupicapra rupicapra*) populations of the southern and northern Pre-Alps (Rughetti & Festa-Bianchet 2012; Willisch *et al.* 2013). Variable responses among different climatic regions seem to be a common pattern. Different effects of the NAO on similar chamois and isard species (*R. rupicapra* and *R. pyrenaica*) were observed in the Alps and Pyrenees (Loison *et al.* 1999). Comparing the same large herbivore (red deer) in contrasting areas across wide latitudinal ranges revealed different seasonal limitations and climatic (NAO) influences on body growth (Martínez-Jauregui *et al.* 2009). Hibernating species such as marmots were negatively affected by climate warming in the European Alps (Tafani *et al.* 2013), but positively in the Upper East River Valley, Colorado, USA (Ozgul *et al.* 2010), due to different environmental constraints, including climate.

Annual population growth of 26 Swiss ibex populations was heterogeneous and not well synchronised, apparently due to large spatial heterogeneity in the effects of various meteorological parameters in different populations (Grøtan *et al.* 2008). Effects of synoptic patterns additionally diminish at smaller scales, where complex topography further hampers the process of climatological downscaling, which subsequently may yield variation in local weather conditions leading to the break-up of synchrony of ecological responses even at quite small spatial scales that are generally characterised by similar seasonal limitations (Pettorelli *et al.* 2005). Differences between sympatric species in their responses to climatic fluctuations remain poorly understood. Ungulate species with contrasting life histories and feeding habits such as wild boar and roe deer exhibited interspecific synchrony in Poland (Mysterud *et al.* 2007). Severe winter conditions, especially rain-on-snow events may synchronise large regions and several rodent species (Kausrud *et al.* 2008) or even an entire mammal community such as under the extreme conditions on Svalbard (Stien *et al.* 2012; Hansen *et al.* 2013).

Our results highlight the significance of the environment in general, and spring weather and snow melt in particular, in determining annual Alpine ibex horn growth rates. Given the limited time-scale of the contemporary shift in ibex morphology, it is likely mainly plastic rather than genetic in origin. Evidence for relatively fast genetic changes (Coltman *et al.* 2003), however, suggests a more careful assessment aiming to disentangle phenotypic plasticity from genetic adaptation that will require additional data (such as genealogical data with pedigrees). Ibex are relatively mobile and thus have the potential to buffer warming by moving up in altitude or change their aspect exposure within a well-defined home range. With an average altitude of 2100 m asl and a considerable amount of Alpine pastures above the upper treeline, the study regions offers ample space for plastic seasonal behaviour patterns. Indeed, the average elevation of locations at which male ibex were shot, slightly increased from 2500 to 2533 m asl, suggesting that animals may behaviourally adapt to changing climates. The role of declining snow cover for herbivores appears a critical issue (Tafani *et al.* 2013), being more likely to affect populations at lower altitudes. The positive association of ibex performance with temperature might result not only from the earlier onset of vegetation development, but also from prolonged optimal feeding conditions during summer via movement to higher elevations where plant phenology is delayed. In the case of predicted continuous decadal-long warming, evolutionary adaptation might be necessary in order to track the speed and magnitude of anthropogenic climate change (Hoffmann & Sgrò 2011). A sound assessment aiming to disentangle phenotypic plasticity from genetic adaptation will require further investigations with data from large mammalian species from different elevational zones or living under various energetic restrictions.

Adaptive capacity might indeed be highest for species living close to their physiological margin, such as the yellow-bellied marmot, a hibernating mammal inhabiting subalpine habitats (Ozgul *et al.* 2010). However, natural selection may not account for changes observed here in long-lived Alpine ibex populations given their reduced gene pool (Biebach & Keller 2009). After near extinction in the 19th century when only *c*. 100 animals survived in the region of the current National Park ‘Grand Paradiso’ in Italy, 88 individuals from this ancestral population were used in a breeding programme to re-introduce ibex to the Swiss Alps during the early 20th century (Biebach & Keller 2012), and only 45 animals were used for the re-settlement in the Canton Grison (Ratti 1994). Population sizes of the eight colonies studied here continuously increased until 1977, after which enactment of strict game regulations resulted in more stable abundances until present (www.jagd-fischerei.gr.ch).

A more careful assessment aimed at disentangling phenotypic plasticity from genetic adaptation will require additional data and should be subject to future studies. Moreover, human hunting could also drive selection for phenotypic trait development. Selective hunting could result in directional changes of reduced horn size over time (Coltman *et al.* 2003; Pérez *et al.* 2011). If hunters are indeed selective, ibex shot at young ages should have longer increments than those shot at later ages. Today, hunting is directed towards both sexes and all age classes. The hunting season is further restricted to the first 3 weeks of October, at which time of the year horn growth is nearly complete (Figure S11). This has resulted in an age-class distribution that mimics natural mortality patterns much better than in most other wildlife species for which hunting selection on life history traits and its evolutionary consequences are documented. Nevertheless, there is a tendency for individuals with slow early growth to generally be shot at later stages than individuals with high early growth rates (unpublished results), as found in other hunted alpine ungulate populations (Coltman *et al.* 2003; Hengeveld & Festa-Bianchet 2011).

Currently, any potential decline in horn growth due to selective harvesting might be masked by the stronger positive effect of increased spring temperature. Therefore, comparative studies with horn collections from non-hunted populations within the same eco-climatic envelop would be useful to assess the harvest-induced selection pressure on life history traits of Alpine ibex populations.
