# Supplementary material for: European springtime temperature synchronises ibex horn growth across the eastern Swiss Alps
Source: Ecol Lett. 2013 Dec 16;17(3):303–13. doi: 10.1111/ele.12231 (PMC4257578; doi:10.1111/ele.12231)
Supplement: Supplementary file 1 — Supplementary [file ele0017-0303-SD1.docx]

Supporting Information (Figures S1-S11 and Tables S1-S3)

**European springtime temperature synchronizes ibex horn growth across the eastern Swiss Alps**

Ulf Büntgen,1,2,3* Andrew Liebhold,4 Hannes Jenny,5 Atle Mysterud,6 Simon Egli,1 Daniel Nievergelt,1 Nils C. Stenseth,6 and Kurt Bollmann1

*1Swiss Federal Research Institute for Forest Snow and Landscape (WSL), CH-8903 Birmensdorf, Switzerland*

*2Oeschger Centre for Climate Change Research (OCCR), University of Bern, CH-3012 Bern, Switzerland*

*3Global Change Research Centre AS CR, v.v.i., Bělidla 986/4a, CZ-60300 Brno, Czech Republic*

*4Northern Research Station, USDA Forest Service, Morgantown, WV-26505, USA*

*5Department of Wildlife and Fishery Service Grison, CH-7001 Chur, Switzerland*

*6Centre for Ecological and Evolutionary Synthesis (CEES), Department of Biosciences, University of Oslo, PO Box NO-1066, Blindern, 0316 Oslo, Norway*


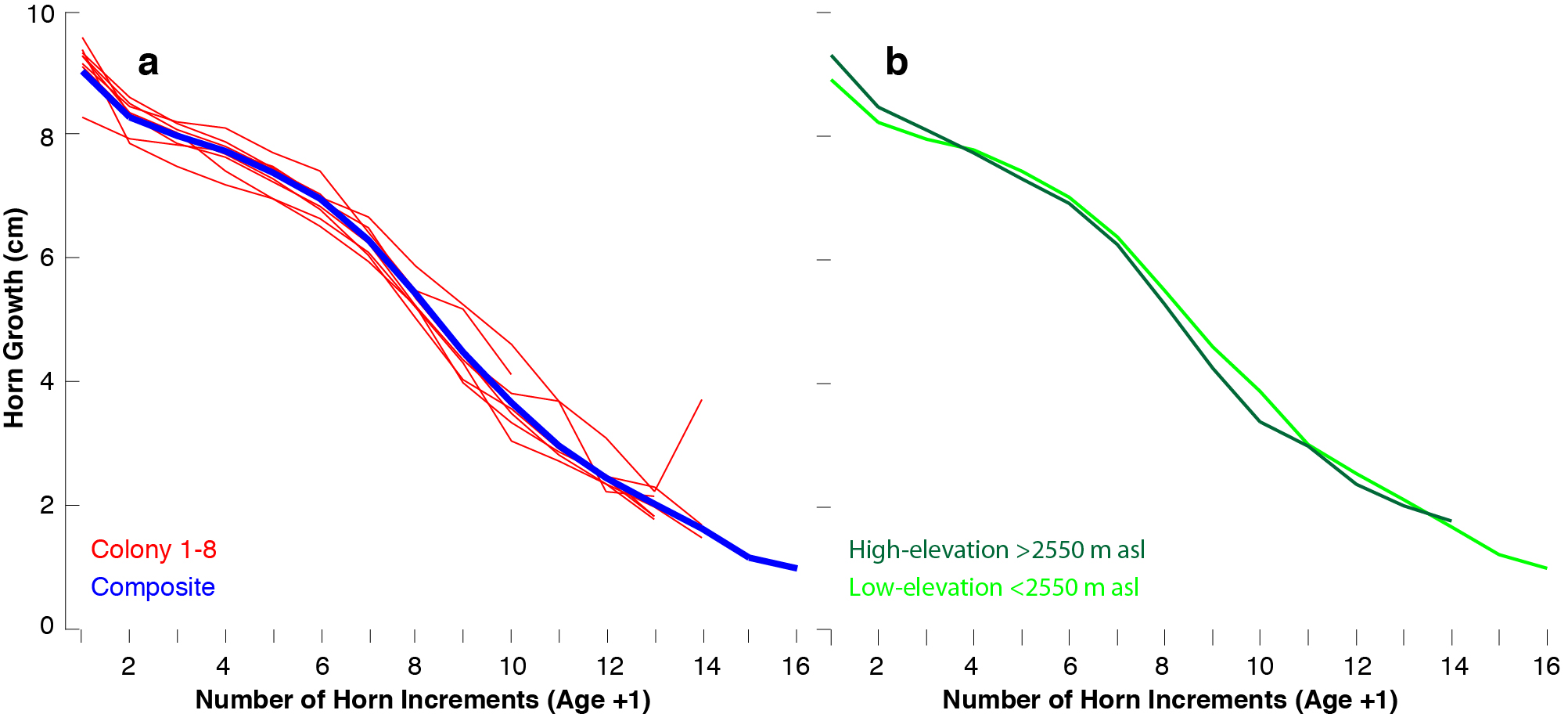


**Figure S1 | Age-trend.** (**a**) Mean growth trends of the age-aligned horn series that were independently computed for the eight disjunct colonies (red), as well as their composite (grand average mean; blue), following methods described in Esper *et al.* (2003). The so-called Regional Curves (RCs) that originate as a result from the Regional Curve Standardization (RCS; Becker et al. 1989) method have been truncated at a minimum replication of ten horns (outermost right-side). (**b**) Regional curves (RCs) separately computed for two high- and low-elevation data subsets (>/<2’550m asl). Both figure parts emphasize the overall high level of coherency among growth levels and trends, indispensable of the colony and/or elevation subset used. That is, horn growth (i.e. the length of annual increments) occurs on a similar level in each of the eight colonies and also when averaging over different elevations. All timeseries smoothing was performed by means of cubic spline functions (Cook & Peters 1981), and variance stabilization followed methods described in Osborn *et al.* (1997).


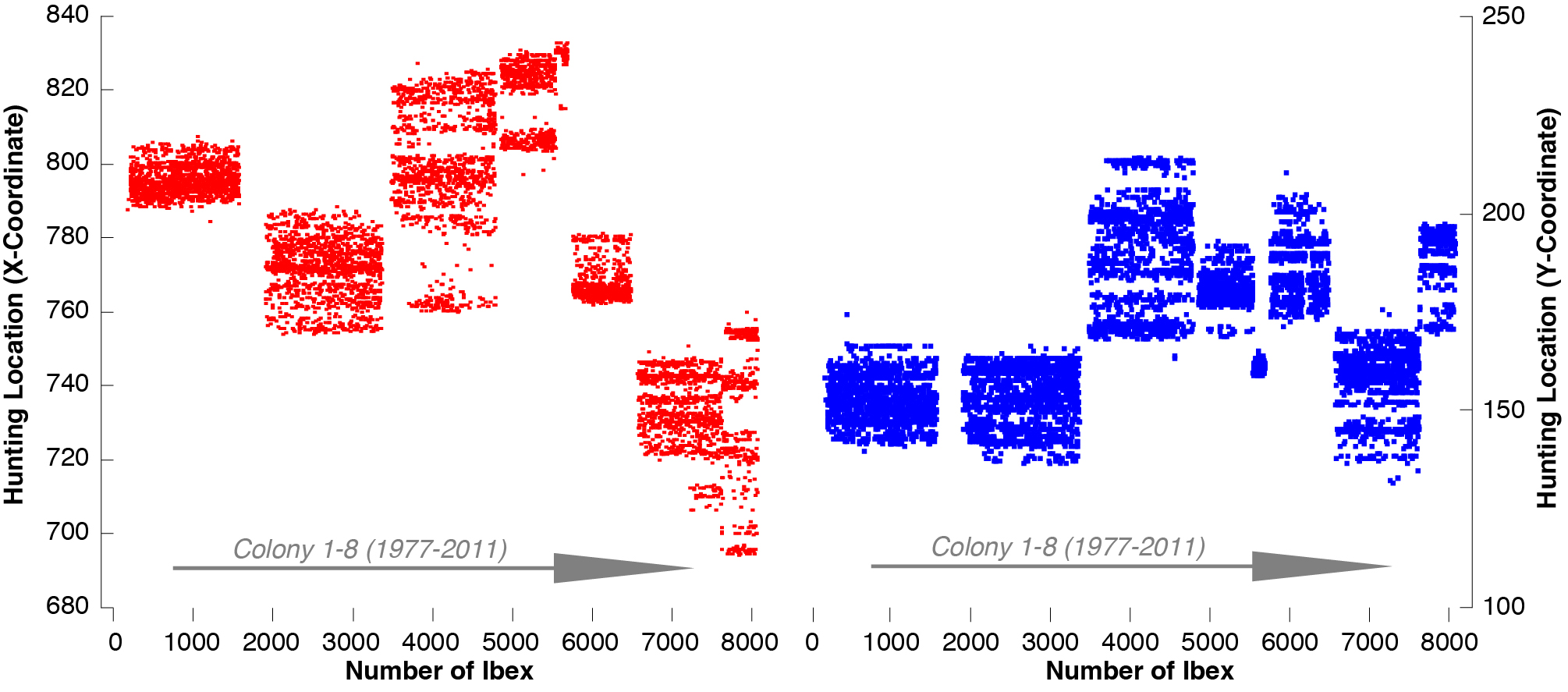


**Figure S2 | Age-dependent horn growth variability.** Spatial distribution of ibex hunting locations within and between the eight disjunct colonies, with the red and blue dots referring to their corresponding x- and y-coordinates (space synonyms), respectively. Since all 8’043 male Alpine ibex hunting locations follow a strict chronological order, i.e. the first (oldest) dates are on the left and the last (youngest) are on the right, this figure reveals that no temporal changes in hunting location occurred between 1977 and 2011. The eight disjunct ibex populations are therefore assumed to be stationary over time.


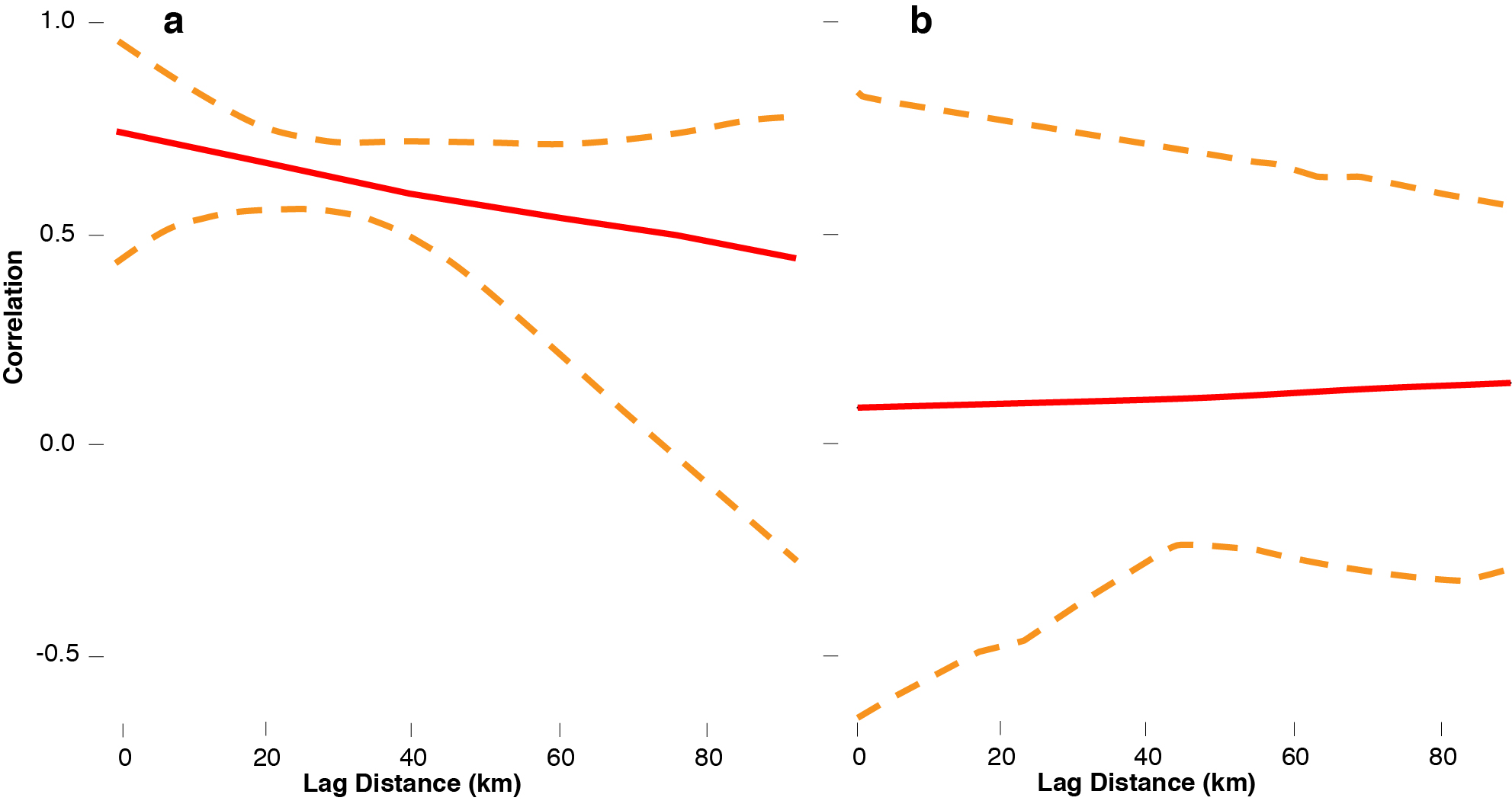


**Figure S3 | Spatial synchrony.** Covariance function of spatial synchrony calculated among (**a**) horn growth as well as (**b**) population size of the eight ibex colonies (solid line), following methods described in Bjørnstad & Falck (2001). Dashed lines show corresponding 95% bootstrap confidence intervals. Results for horn growth indicate significant global synchrony among all colonies (global synchrony of 0.58 with 95% confidence interval of 0.44 to 0.72. Synchrony decreased with increasing distance between populations. Non-significant synchrony was, however, found for changes in population size. All calculations are based on data from the common period 1979-2011.


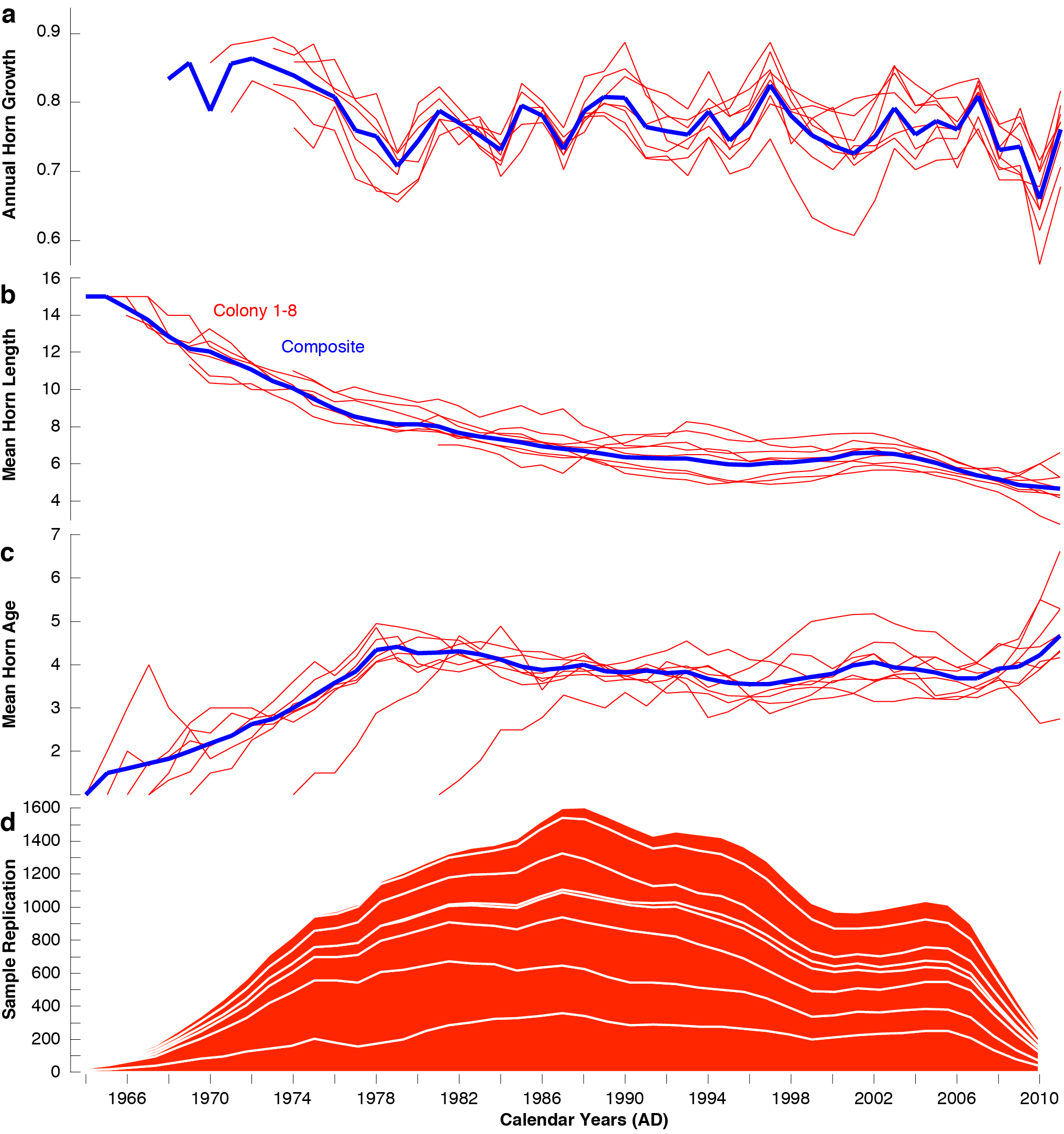


**Figure S4 | Horn chronology characteristics.** (**a**) Raw chronologies of year-to-year horn growth without application of any detrending technique, i.e. correction for age trends. The individual timeseries are truncated at a minimum sample size of 20 horns and the red lines refer to data from the eight individual colonies, whereas the blue line was calculated by means of the pooled dataset including measurements from all 8,043 male Alpine ibex. (**b**) Mean horn length (segments), (**c**) mean horn age (years) computed for the eight individual colonies (red) and their composite (blue). (**d**) Sample replication of the eight individual colonies that must be considered as possible reason for the detection of artificial variance changes during periods of less replication.

**
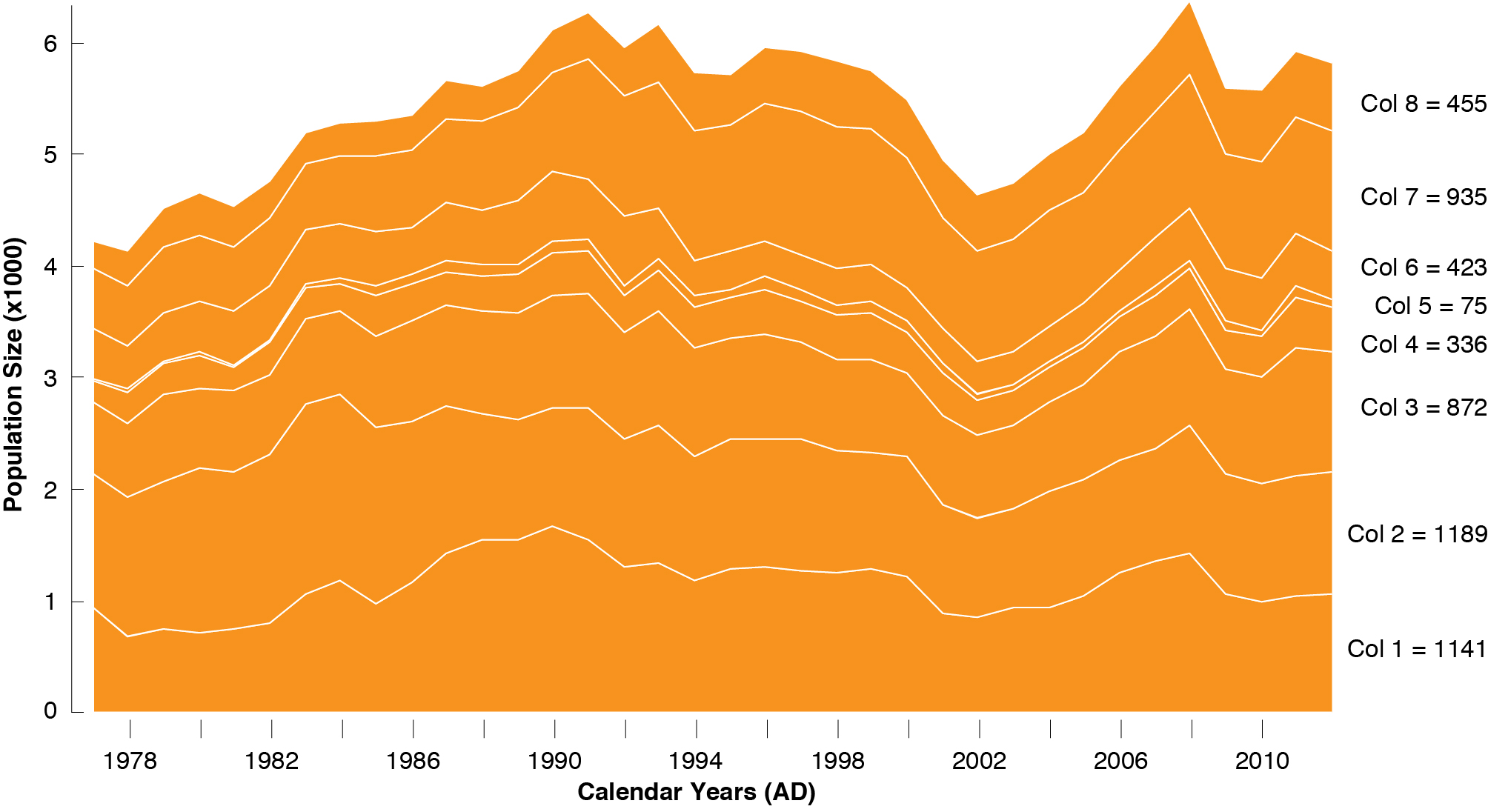
**

**Figure S5 | Population size.** Colony-specific estimates of population size, with the right-side values referring to the corresponding long-term means that were calculated for the 1976-2012 period.

**
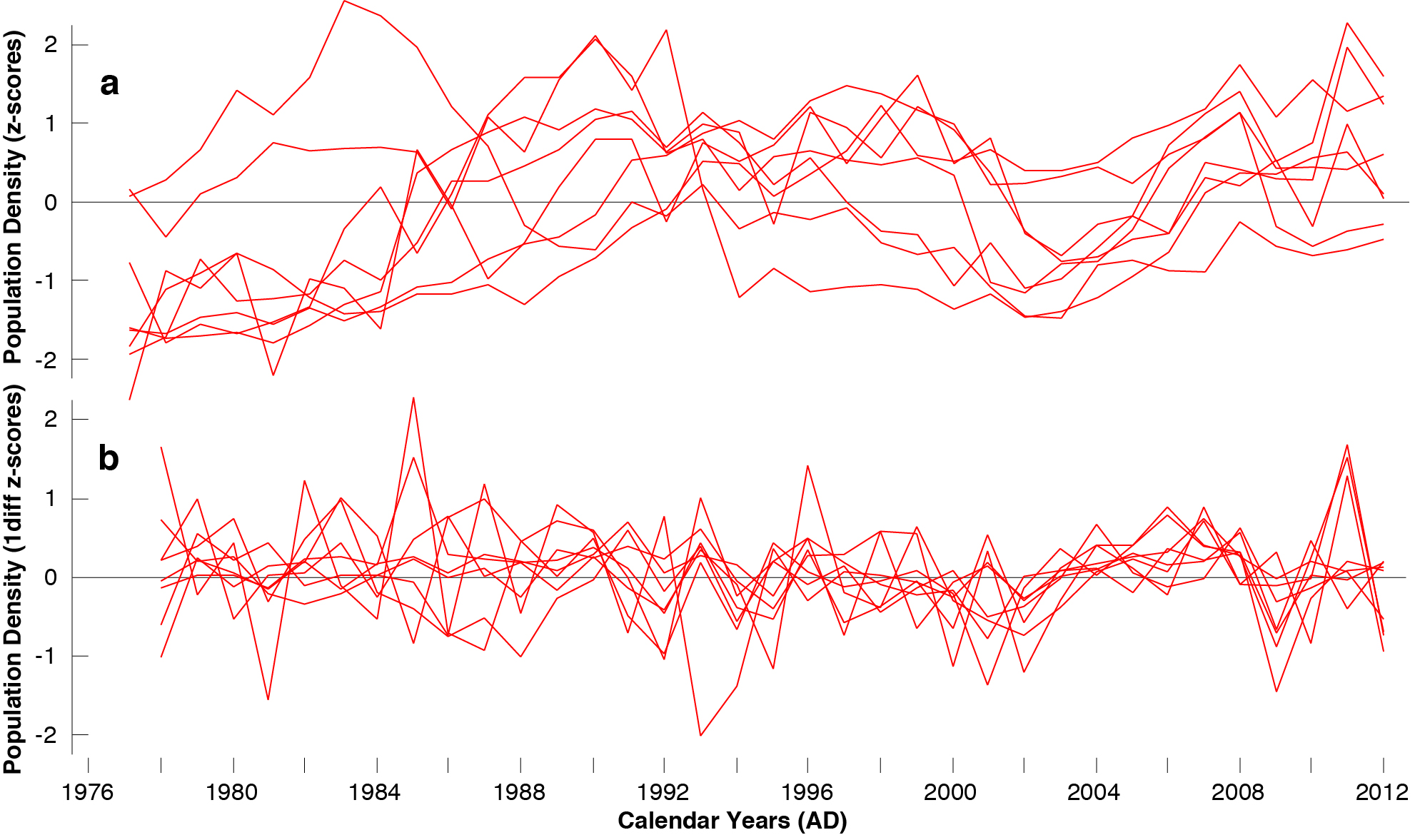
**

**Figure S6 | Population size.** (**a**) Changes in population size of the eight ibex colonies after normalization (mean of zero and standard deviation of one). (**b**) The first difference of the same timeseries to emphasize year-to-year variability rather than longer-term trends.


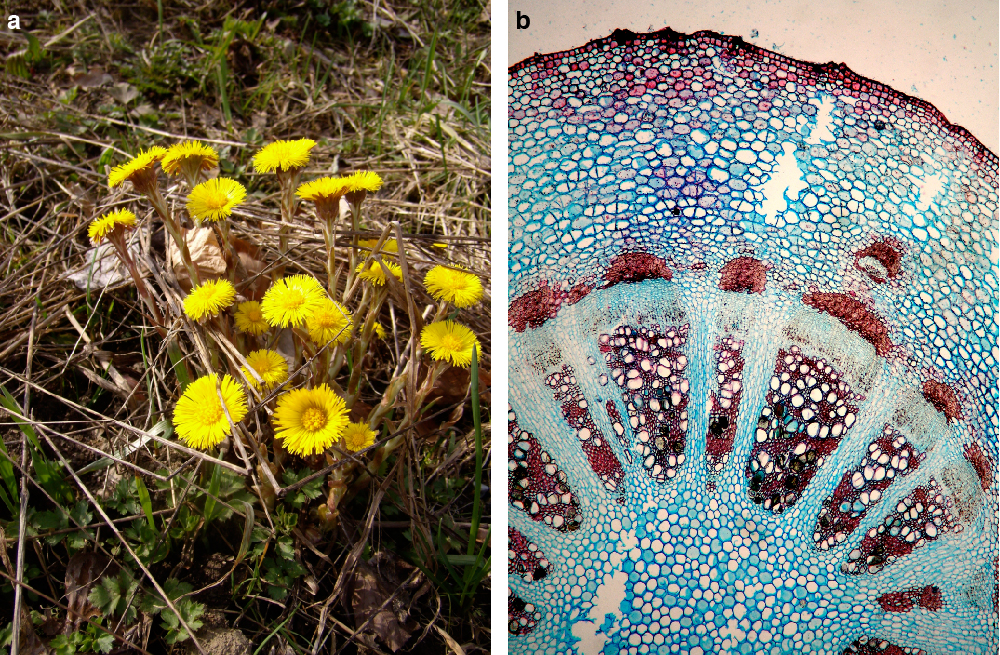


**Figure S7 | Plant phenology. a**, Macroscopic morphological and **b**, microscopic anatomical perspective on the Alpine coltsfoot (*Tussilago farfara*), which has been identified as the best plant phenological indicator to explain ibex horn growth variability.


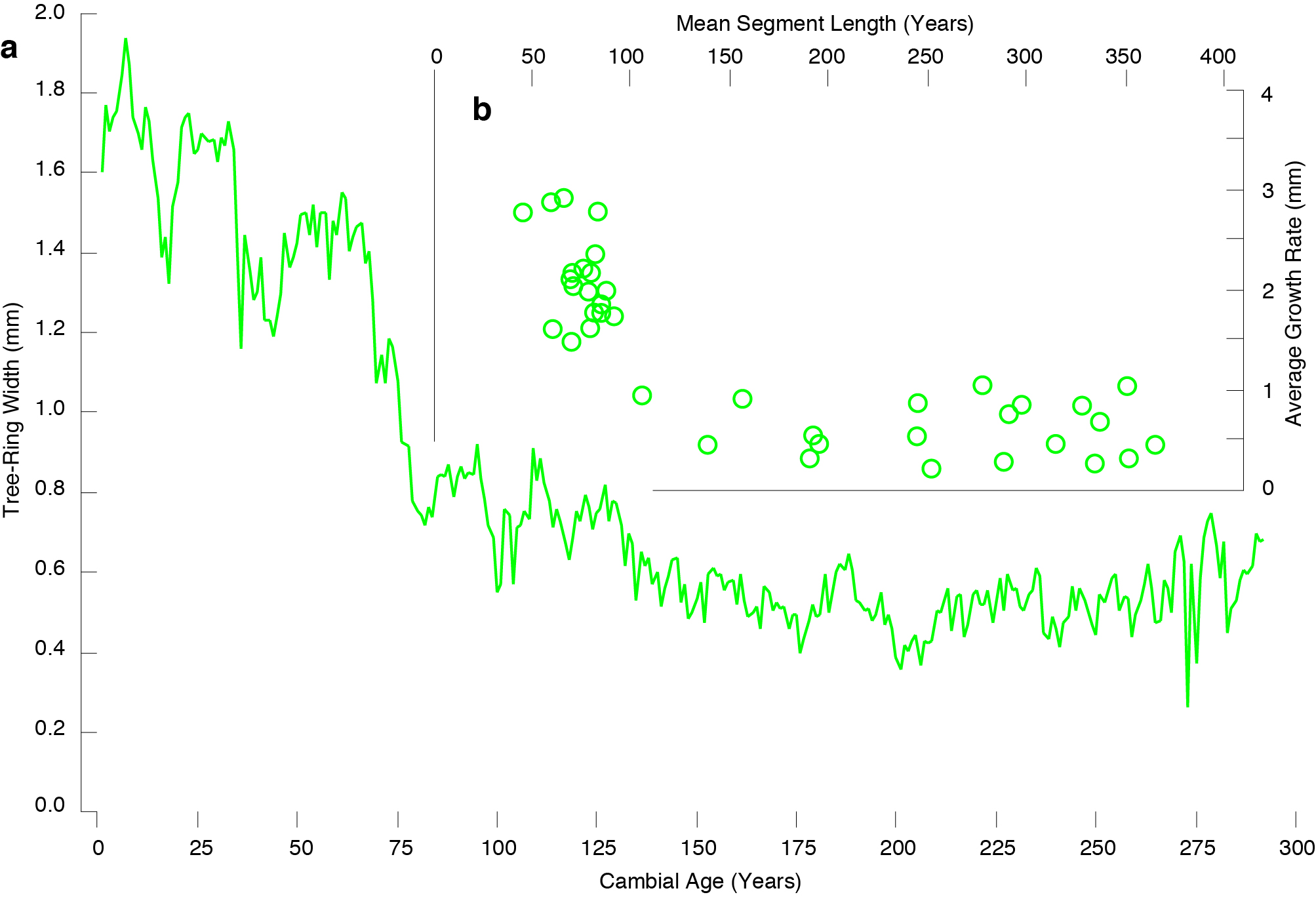


**Figure S8 | Tree growth trend.** (**a**) Mean growth trend of the age-aligned tree-ring width series from 42 high-elevation pine (*Pinus cembra*) trees that were samples in the eastern Swiss Alps. This so-called Regional Curves (RCs) that originates as a result from the Regional Curve Standardization (RCS) method has been truncated at a minimum replication of ten series. The RC nicely describes the shape of a negative exponential function. (**b**) The relationship between mean segment length (MSL) and average growth rate (AGR) of the 42 pine trees.


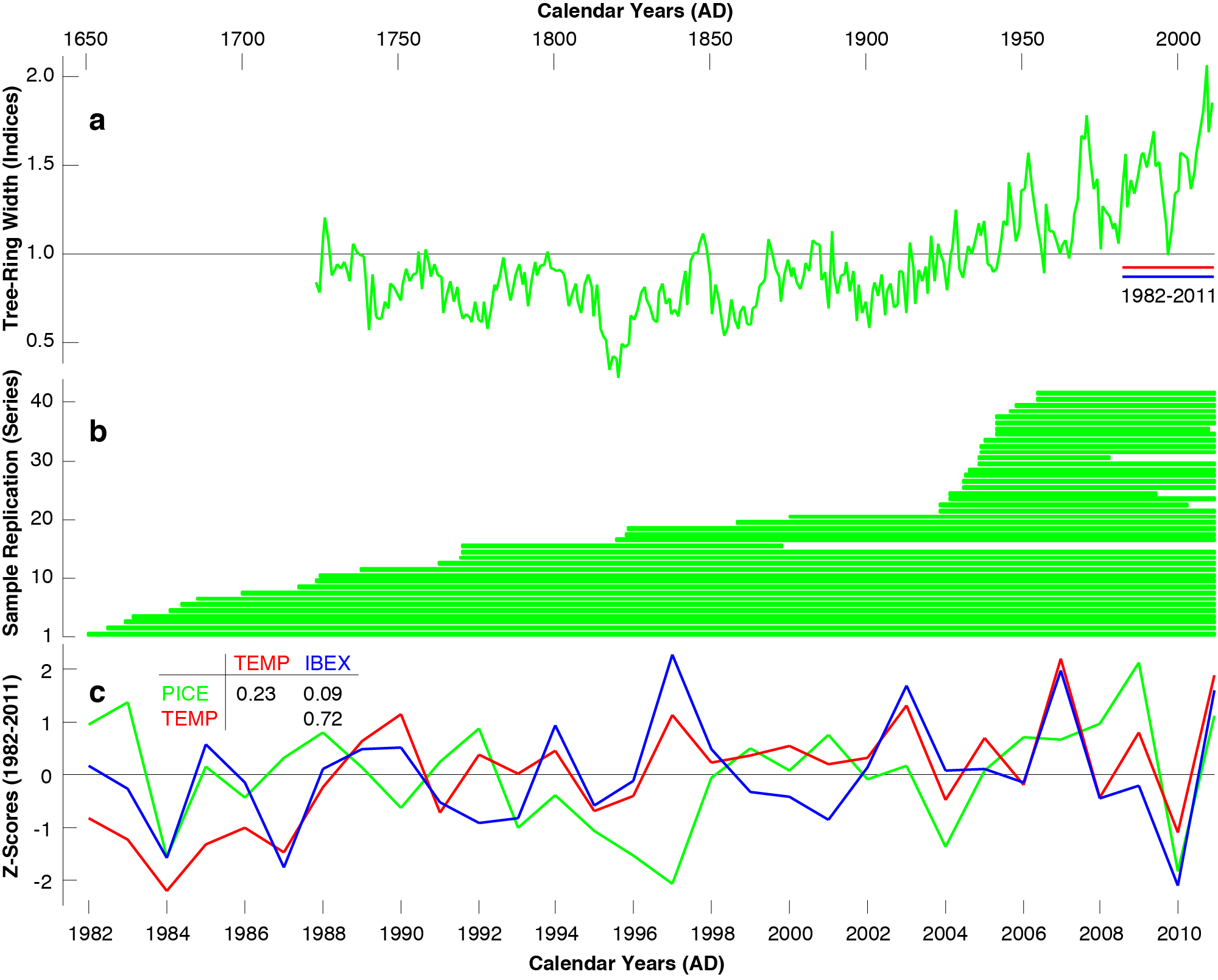


**Figure S9 | Tree growth variation.** (**a**) Mean pine ring width chronology after RCS detrending, with the red and blue horizontal bars indicating the rather short period of overlap between tree growth, horn growth and temperature readings. (**b**) Replication of the pine dataset with each bar referring to one specimen. (**c**) Comparison between tree and horn growth as well as March-May temperature.


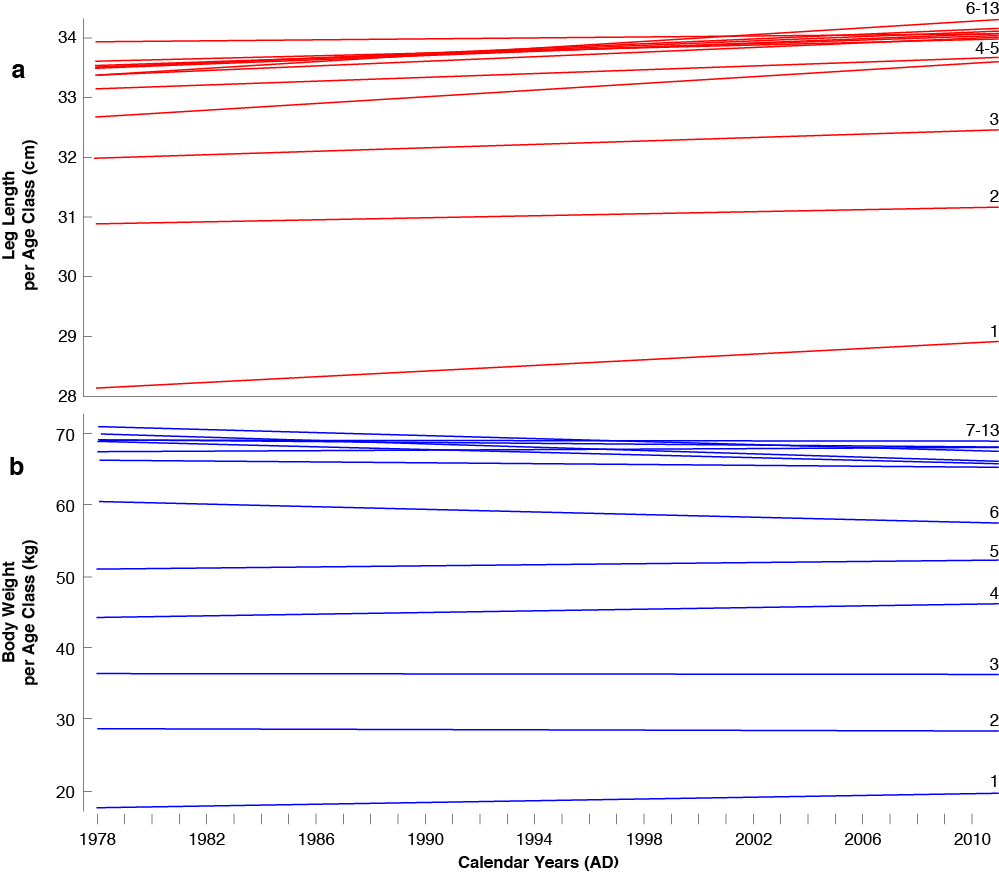


**Figure S10 | Phenotypic capacity.** (**a**) Mean annual hind foot length (cm), and (**b**) mean annual body weight (kg) of each individual male ibex computed over two early/late split periods (1978-1994/1995-2011) and per age classes for with >100 horn series were available (1-13). Horizontal bars refer to mean values associated with the +/- 1 standard error.


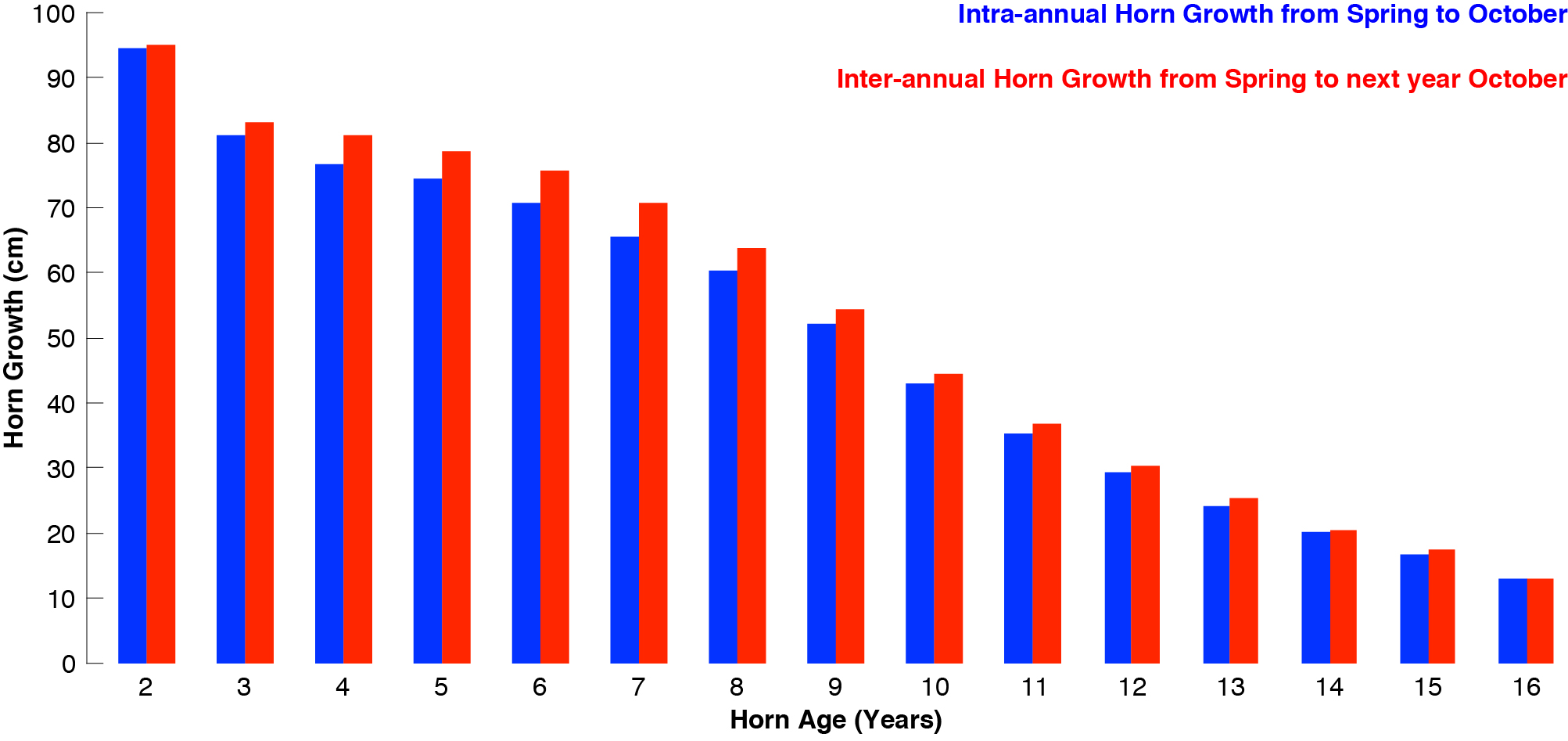


**Figure S11 | Horn growth termination.** Comparison of age-specific annual growth rates computed for animals that were hunted within the first weeks of October in the year of horn formation (blue) and for animals that were hunted in following years (red), i.e. at older ages.

**Table S1 | Model performance.** Results from model selection on a subset of the data (years 1976-2011) for which population size data were available. The identity of each individual was entered as a random term in all models. We report the Akaike Information Criterion (AIC) and differences (ΔAIC) thereof between each model and the model with the lowest AIC 1 indicates variables included into the model.

| **longevity** | **colony** | **Colony:age** | **Colony:(age)2** | **Colony:(age)3** | **ln(population size)** | **mamTmax** | **snow35** | **Year (as trend)** | **colony:ln(population size)** | **colony :mamTMax** | **colony :snow35** | **colony :year** | **AIC** | **ΔAIC** |
| --- | --- | --- | --- | --- | --- | --- | --- | --- | --- | --- | --- | --- | --- | --- |
| 1 | 1 | 1 | 1 | 1 | 1 | 1 | 1 | 1 | 1 | 1 | 1 | 1 | -12950.4 | 263.1 |
| 1 | 1 | 1 | 1 | 1 | 1 | 1 | 1 | 1 | 1 | 1 | 1 |  | -12991.7 | 221.8 |
| 1 | 1 | 1 | 1 | 1 | 1 | 1 | 1 | 1 | 1 | 1 |  |  | -13089.7 | 123.8 |
| 1 | 1 | 1 | 1 | 1 | 1 | 1 | 1 | 1 | 1 |  |  |  | -13147.9 | 65.6 |
| 1 | 1 | 1 | 1 | 1 | 1 | 1 | 1 |  | 1 |  |  |  | -13050.2 | 163.2 |
| 1 | 1 | 1 | 1 | 1 | 1 | 1 |  | 1 | 1 |  |  |  | -12993.6 | 219.9 |
| 1 | 1 | 1 | 1 | 1 | 1 | 1 | 1 | 1 |  |  |  |  | -13127.1 | 86.4 |
| 1 | 1 | 1 | 1 | 1 |  | 1 | 1 | 1 |  |  |  |  | -13136.5 | 76.9 |
| 1 | 1 | 1 | 1 |  | 1 | 1 | 1 | 1 | 1 |  |  |  | -13213.5 | 0 |
| 1 | 1 | 1 |  |  | 1 | 1 | 1 | 1 | 1 |  |  |  | -12928.7 | 284.8 |
| 1 | 1 |  |  |  | 1 | 1 | 1 | 1 | 1 |  |  |  | -11793.6 | 1419.8 |
| 1 | 1 | 1 | 1 |  | 1 | 1 | 1 | 1 | 1 |  |  |  | -12820.6 | 392.8 |

**Table S2 | Model trials.** Parameter estimates from the best model in Table S1. The identity of each individual was entered as a random term. Note that baseline is for colony 1.

| **Parameter** | **Estimate** | **Std. Error** | **Lower 95%** | **Upper 95%** |
| --- | --- | --- | --- | --- |
| **Intercept** | 8.42 | 0.4054 | 7.6092 | 9.2308 |
| **age** | 0.0136 | 0.0046 | 0.0044 | 0.0227 |
| **(age)2** | -0.001 | 0.0006 | -0.0022 | 0.0002 |
| **(age)3** | -0.0005 | 0 | -0.0005 | -0.0004 |
| **longevity** | -0.0007 | 0.0003 | -0.0012 | -0.0002 |
| **Colony 2 vs. 1** | 0.0335 | 0.1485 | -0.2635 | 0.3305 |
| **Colony 3 vs. 1** | 1.404 | 0.1699 | 1.0642 | 1.7438 |
| **Colony 4 vs. 1** | 0.9532 | 0.1599 | 0.6334 | 1.273 |
| **Colony 5 vs. 1** | 0.7346 | 0.1485 | 0.4376 | 1.0316 |
| **Colony 6 vs. 1** | 0.2725 | 0.1335 | 0.0055 | 0.5395 |
| **Colony 7 vs. 1** | 0.4859 | 0.1242 | 0.2375 | 0.7343 |
| **Colony 8 vs. 1** | 0.434 | 0.1837 | 0.0666 | 0.8014 |
| **Max temp (Mar-May)** | 0.024 | 0.0011 | 0.0218 | 0.0261 |
| **log(population size)** | 0.0297 | 0.0111 | 0.0076 | 0.0518 |
| **Snow cover (Mar-May)** | -0.0002 | 0 | -0.0002 | -0.0002 |
| **Year as trend** | -0.0022 | 0.0002 | -0.0026 | -0.0018 |
| **Colony 2 vs. 1: age** | -0.0683 | 0.0041 | -0.0764 | -0.0601 |
| **Colony 3 vs. 1: age** | -0.0879 | 0.0044 | -0.0966 | -0.0791 |
| **Colony 4 vs. 1: age** | -0.0943 | 0.005 | -0.1042 | -0.0844 |
| **Colony 5 vs. 1: age** | -0.0761 | 0.0109 | -0.0979 | -0.0543 |
| **Colony 6 vs. 1: age** | -0.105 | 0.0053 | -0.1155 | -0.0945 |
| **Colony 7 vs. 1: age** | -0.0563 | 0.0048 | -0.0658 | -0.0467 |
| **Colony 8 vs. 1: age** | -0.0706 | 0.0065 | -0.0835 | -0.0576 |
| **Colony 2 vs. 1: (age)2** | 0.0029 | 0.0003 | 0.0023 | 0.0035 |
| **Colony 3 vs. 1: (age)2** | 0.0044 | 0.0003 | 0.0037 | 0.005 |
| **Colony 4 vs. 1: (age)2** | 0.0058 | 0.0003 | 0.0051 | 0.0065 |
| **Colony 5 vs. 1: (age)2** | 0.0042 | 0.0008 | 0.0025 | 0.0058 |
| **Colony 6 vs. 1: (age)2** | 0.0048 | 0.0004 | 0.004 | 0.0055 |
| **Colony 7 vs. 1: (age)2** | 0.0018 | 0.0004 | 0.0011 | 0.0025 |
| **Colony 2 vs. 1: (age)2** | 0.0038 | 0.0005 | 0.0029 | 0.0048 |
| **Colony 2 vs. 1:log(population size)** | 0.0306 | 0.0208 | -0.0111 | 0.0722 |
| **Colony 3 vs. 1:log(population size)** | -0.1633 | 0.0249 | -0.213 | -0.1136 |
| **Colony 4 vs. 1:log(population size)** | -0.1165 | 0.0264 | -0.1692 | -0.0638 |
| **Colony 5 vs. 1:log(population size)** | -0.0927 | 0.03 | -0.1526 | -0.0328 |
| **Colony 6 vs. 1:log(population size)** | 0.016 | 0.0208 | -0.0255 | 0.0575 |
| **Colony 7 vs. 1:log(population size)** | -0.0375 | 0.0178 | -0.073 | -0.0019 |
| **Colony 8 vs. 1:log(population size)** | -0.0236 | 0.029 | -0.0817 | 0.0344 |

**Table S3 | Population size. a**, Pearson’s correlation coefficients between the eight individual population size timeseries computed over the 1983-2012 robust period. **b**, Pearson’s correlation coefficients based on the first difference of the same timeseries to capture year-to-year relationships rather than longer-term agreement. Grand average means are provided at the right side, and correlations with the corresponding horn growth chronologies are indicated at the bottom.

|  | **Col 1** | **Col 2** | **Col 3** | **Col 4** | **Col 5** | **Col 6** | **Col 7** | **Col 8** |  |
| --- | --- | --- | --- | --- | --- | --- | --- | --- | --- |
| **a** |  | 0.06 | 0.44 | 0.10 | 0.58 | 0.52 | 0.08 | -0.28 | **Col 1** |
|  |  |  | -0.18 | -0.37 | -0.21 | 0.37 | -0.67 | -0.67 | **Col 2** |
|  |  |  |  | 0.53 | 0.51 | 0.43 | 0.33 | 0.36 | **Col 3** |
|  |  |  |  |  | 0.65 | -0.15 | 0.64 | 0.54 | **Col 4** |
|  |  |  |  |  |  | 0.13 | 0.44 | 0.12 | **Col 5** |
|  |  |  |  |  |  |  | -0.48 | -0.45 | **Col 6** |
|  |  |  |  |  |  |  |  | 0.82 | **Col 7** |
|  |  |  |  |  |  |  |  |  | **Col 8** |
|  | -0.03 | -0.14 | 0.08 | 0.17 | 0.10 | -0.08 | 0.01 | -0.01 |  |
|  |  |  |  |  |  |  |  |  |  |
|  | **Col 1** | **Col 2** | **Col 3** | **Col 4** | **Col 5** | **Col 6** | **Col 7** | **Col 8** |  |
| **b** |  | 0.19 | 0.35 | -0.09 | 0.09 | 0.14 | 0.40 | 0.04 | **Col 1** |
|  |  |  | 0.09 | 0.04 | -0.05 | -0.02 | 0.18 | 0.18 | **Col 2** |
|  |  |  |  | 0.55 | 0.55 | 0.05 | 0.20 | 0.16 | **Col 3** |
|  |  |  |  |  | 0.61 | -0.05 | 0.07 | 0.01 | **Col 4** |
|  |  |  |  |  |  | -0.05 | 0.24 | -0.11 | **Col 5** |
|  |  |  |  |  |  |  | -0.15 | -0.02 | **Col 6** |
|  |  |  |  |  |  |  |  | 0.47 | **Col 7** |
|  |  |  |  |  |  |  |  |  | **Col 8** |
|  | 0.04 | -0.01 | 0.23 | 0.40 | 0.38 | -0.02 | 0.11 | -0.07 |  |

**References**

Becker, M., Landmann, G. & Levy, G. (1989). Silver fir decline in the Vosges Mountains (France): Role of climate and silviculture. Water, Air, Soil Poll., 48, 77–86.

Bjørnstad, O.N. & Falck, W. (2001). Spatial covariance functions: estimation and testing. Environ. Ecol. Statistics, 8, 53–70.

Cook, E.R. & Peters, K. (1981). The smoothing spline: A new approach to standardizing forest interior tree-ring width series for dendroclimatic studies. Tree-Ring Bulle., 41, 45–53.

Esper, J., Cook, E.R., Krusic, P.J., Peters, K. & Schweingruber, F.H. (2003). Tests of the RCS method for preserving low-frequency variability in long tree-ring chronologies. Tree-Ring Res., 59, 81–98.

Osborn, T.J., Briffa, K.R. & Jones, P.D. (1997). Adjusting variance for sample-size in tree-ring chronologies and other regional-mean time-series. Dendrochronologia, 15, 89–99.
